# Emergency surgery in a severe penetrating skull base injury by a screwdriver: case report and literature review

**DOI:** 10.1186/1749-7922-1-36

**Published:** 2006-12-14

**Authors:** Antonio De Tommasi, Pasquale Cascardi, Claudio De Tommasi, Sabino Luzzi, Pasqualino Ciappetta

**Affiliations:** 1 Department of Neurology and Psychiatry Sciences, Chair of Neurosurgery, University of Bari, 70124 Bari, Italy

## Abstract

**Background:**

Very few cases of severe penetrating injuries to the skull base with a seemingly innocuous object have been described in the literature. Of the cases reported, only ten involve a penetrating screwdriver. However, the choice of therapeutic management, whether it be emergency surgical or non-surgical removal of the penetrating object as well as the selected surgical approach remain quite controversial.

**Case presentation:**

The authors describe the case of a severe penetrating skull base injury caused by a screwdriver, following an accidental fall from a ladder. The patient was admitted in Glasgow Coma Scale (GCS) 11 with a cerebrospinal fluid (CSF) leak in the right maxillary area. The tri-dimensional computerized tomography (3-D CT) scan revealed an oblique trajectory of the screwdriver shank through the skull base. The authors opted for an emergency surgical extraction of the object. A contra-lateral pterional approach was successfully performed and a two-year follow-up showed no neurological deficits.

**Conclusion:**

The reported case supports the choice of emergency surgical removal of the object in penetrating skull base injuries involving the anterior skull base with neurovascular lesions. Surgical aspects of the pterional approach, and in particular the left pterional approach as well as other cranio-facial approaches in severe penetrating skull base injuries are discussed.

## Background

Severe penetrating head injuries caused by pointed objects account for about 0.4% of all head injuries [1-3]. The literature reports ten cases wherein the penetrating object was a screwdriver [4-10]. In five of these cases, the patient survived and in four of these five cases, the patient presented either anatomical or functional deficits [2,8,10] (Table 1).

**Table 1:** Review of penetrating skull base injuries caused by screwdriver

***Author***	***Cases***	***Cause***	***Therapy***	***Outcome***	***Comments***
**Smrkolj V. 1995**	two	Homicidal attempt	Surgery	Died	Left hemiplegia, right internal carotid artery thrombosis
		Suicidal attempt	Surgery	Died	Brain edema and right anterior cerebral artery infarction
**Anderson S.A. 1996**	one	Suicidal attempt	No surgery	Survived	Right orbital injury No deficits
**Evans R.J. 1996**	one	Accident	No surgery	Died	Hard palate impalement
**Li T. 2000**	one	Homicidal attempt	No surgery	Died	Two weeks later: basilar-cavernous fistula and basilar artery aneurysm
**Tutton M.G. 2000**	four	Homicidal attempt	No surgery	Survived	Left parietal hemorrhage involving lateral ventricles; right hemiplegia, mild dysphasia
			No surgery	Survived	Intracerebral haematoma in the left frontal lobe with an overlying skull vault fracture
			Surgery	Died	Depression of the left temporal fossa with acute sub-dural hemorrhage (died two days after surgery)
			No Surgery	Died	Tip of the screwdriver penetrated into the brain stem
**Wong S.C. 2002**	one	Accident	No surgery	Survived	Traumatic optic neuropathy
**De Tommasi A. 2006**	one	Accident	Surgery	Survived	No deficits

The present paper reports the case of a penetrating skull base injury patient who, despite the severity of the injury, survived with no neurological deficits. This is owing to the choice of an emergency surgical removal of the penetrating object.

## Case presentation

**Figure 1:**
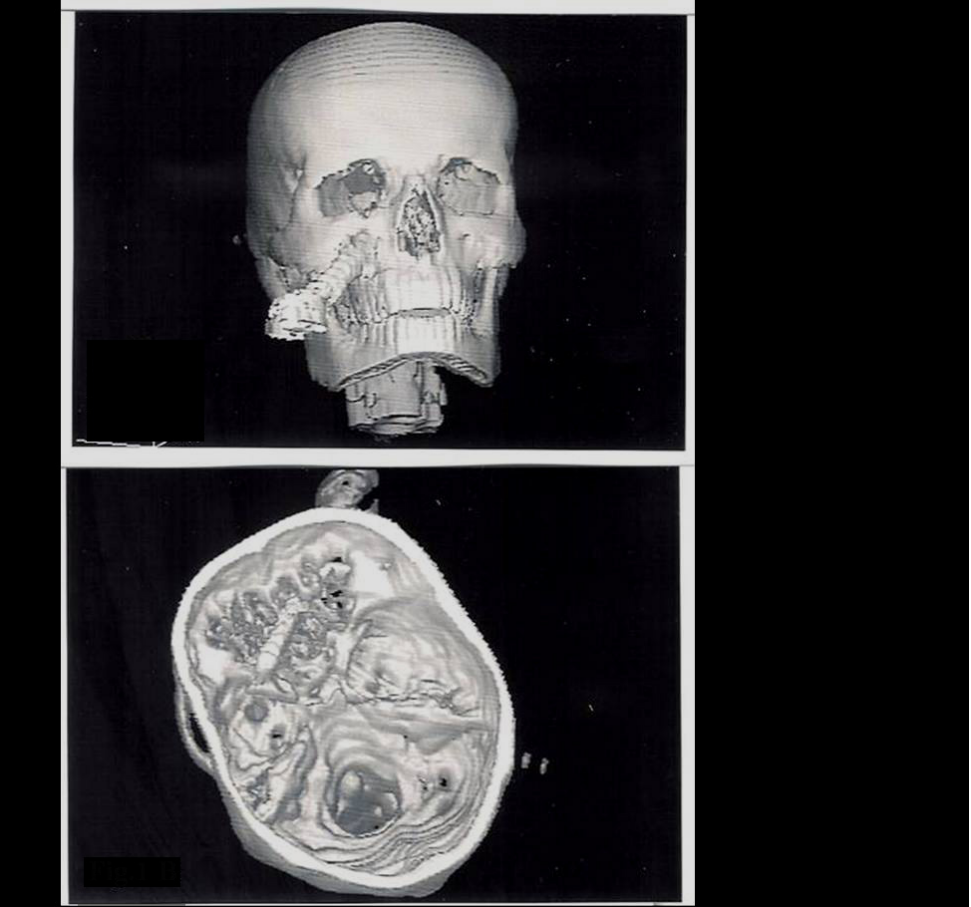
A 3-D CT scan revealed the severity of the skull base penetrating injury.

A 20 year-old male suffered a severe penetrating skull base injury caused by a screwdriver which he was holding in his right hand when he accidentally slipped off a ladder. The crossed-head tip of the screwdriver pierced into the right maxilla and the steel shank penetrated through the skull base. The total penetration trajectory measured roughly ten centimeters. The patient was admitted in GCS 11. He presented a scotoma in the left eye and a CSF leak in the maxillary area. The 3-D CT-scan revealed an oblique trajectory of the screwdriver through the right maxilla, into the ethmoidal plane and as far as the left sphenoid wing (Fig. 1).

An emergency left pterional approach surgery was performed. The tip of the screwdriver was lodged just above the left optic nerve and adhered tightly to the intracranial part of the nerve trunk. The tip was detected in the left Sylvian area next to the M1 narrowed tract.

First, the screwdriver tip was carefully detached from the artery and, then, the screwdriver was slowly extracted from the skull base. The dura lacerations were repaired with artificial dura, while the skull base fractures were closed with a fragment of temporal muscle and fibrin glue.

The post-operative course was uneventful. The patient's visual acuity began to improve ten days after surgery and, within six weeks from surgery, his visual acuity was completely restored.

## Discussion and conclusion

Despite the need for emergency brain decompression, non-surgical extraction of the screwdriver was promptly rejected by the authors due to the high risk of a middle cerebral artery and/or optic nerve lesion.

Hence, an emergency surgical removal of the screwdriver was selected to preserve both the middle cerebral artery and the function of the left optic nerve. Moreover, surgery allowed to repair the dura, stop the leaking and avoid possible risks of CSF infection.

*The pterional approach *was selected against other cranio-facial approaches, and, in particular, sub-cranial approaches, considering the position of the screwdriver tip in relation to the involved neurovascular structures. Furthermore, although the screwdriver tip had pierced through the right maxilla, a *left pterional approach *was deemed most suitable as it allowed to obtain a direct view of the left optic nerve injury, to explore the middle fossa and to repair the dura laceration.

A *right-side pterional approach *was discarded as it would not have ensured a full view of the intracranial area where the screwdriver was lodged nor the neurovascular structures by it affected.

Although a *left frontal approach *allows to easily reach the left orbital roof and optic nerve, it could have resulted dangerous for a frontal pole retraction and possibly caused damage to both parenchyma and arteries.

A *left sub-frontal approach to the orbit *might have been suitable if the tip of the screwdriver had caused a fracture of the left orbital roof and/or the left ethmoidal plane with or without optic nerve involvement. Furthermore, as the reported case required exploration of both the frontal pole and the Sylvian fossa, this approach was not deemed suitable.

An *extra-cranial approach to the orbit*, such as the anterior superior approach, anterior median approach and Krönlein approach, would have been appropriate if the screwdriver had involved the left orbit with no anterior skull base fractures.

The *sub-cranial approach*, introduced by Raveh in 1978 and recently emphasized by Schaller [11], was deemed least suitable in the reported case due to the frontal pole involvement and to the anatomical relationship between the screwdriver tip and the left middle cerebral artery. In fact, this approach is not only not advised, but even contraindicated in skull base injuries requiring surgical access to the frontal and/or temporal lobes. Furthermore, this approach may cause complications such as anosmia (especially when a fracture is detected [12], CSF leaks, late enophthalmus and mucocele [11].

It is believed that the choice surgical approach in penetrating skull base injuries must: a) maximize bone and dura exposure, b) allow treatment of associated intracranial injuries, c) avoid sinus opening, d) preserve olfaction, e) minimize surgical stress and f) minimize anti-aesthetic scars.

In respect of the above and on the basis of the anatomical relationship between the screwdriver and the involved neurovascular structures, the authors chose to perform an emergency left pterional approach surgery. The selected surgical approach allowed good cerebral and neurovascular decompression, maximum exposure of the skull base and dura, direct view of the optic nerve injury, exploration of the middle fossa and its vascular structures, opportunity to repair the dura, non-opening of the sinus and preservation of the olfactory sense. In addition, the surgical stress was limited and the cosmetic results were very acceptable. Most importantly, however, in the reported case, the patient survived with no neurological deficits.

In conclusion, the authors suggest that an emergency pterional approach surgery may be the preferred therapeutic management in similar severe penetrating skull base injuries caused by a pointed object.

## Abbreviations

GCS: Glasgow Coma Scale

CSF: Cerebrospinal Fluid

3-D CT: Tri-Dimensional Computerized Tomography

## Competing interests

The author(s) declare that they have no competing interests.

## Authors' contributions

**ADT **: conceived the case report, participated in the surgery as first operator and drafted the manuscript.

**PC **: participated in the surgery and in the literature review.

**CDT **: participated in the clinical management of the patient and in literature review.

**SL **: participated in the clinical management of the patient and in literature review.

**PC **: proofread the manuscript.

All authors have read and approved the final manuscript.
